# Machine learning models predicts risk of proliferative lupus nephritis

**DOI:** 10.3389/fimmu.2024.1413569

**Published:** 2024-06-11

**Authors:** Panyu Yang, Zhongyu Liu, Fenjian Lu, Yulin Sha, Penghao Li, Qu Zheng, Kefen Wang, Xin Zhou, Xiaoxi Zeng, Yongkang Wu

**Affiliations:** ^1^Department of Laboratory Medicine, West China Biomedical Big Data Center, West China Hospital, Sichuan University, Chengdu, China; ^2^Jintang First People’s Hospital, Chengdu, China; ^3^Department of Laboratory Medicine, Sichuan Jinxin Xinan Women’s and Children’s Hospital , Chengdu, China; ^4^Department of Obstetrics, Chengdu Jinjiang Hospital for Women & Children Health, Chengdu, China; ^5^Center for Reproductive Medicine, The Third People’s Hospital of Chengdu, Chengdu, China

**Keywords:** proliferative lupus nephritis, machine learning, kidney biopsy, predictive model, diagnostic marker

## Abstract

**Objective:**

This study aims to develop and validate machine learning models to predict proliferative lupus nephritis (PLN) occurrence, offering a reliable diagnostic alternative when renal biopsy is not feasible or safe.

**Methods:**

This study retrospectively analyzed clinical and laboratory data from patients diagnosed with SLE and renal involvement who underwent renal biopsy at West China Hospital of Sichuan University between 2011 and 2021. We randomly assigned 70% of the patients to a training cohort and the remaining 30% to a test cohort. Various machine learning models were constructed on the training cohort, including generalized linear models (e.g., logistic regression, least absolute shrinkage and selection operator, ridge regression, and elastic net), support vector machines (linear and radial basis kernel functions), and decision tree models (e.g., classical decision tree, conditional inference tree, and random forest). Diagnostic performance was evaluated using ROC curves, calibration curves, and DCA for both cohorts. Furthermore, different machine learning models were compared to identify key and shared features, aiming to screen for potential PLN diagnostic markers.

**Results:**

Involving 1312 LN patients, with 780 PLN/NPLN cases analyzed. They were randomly divided into a training group (547 cases) and a testing group (233 cases). we developed nine machine learning models in the training group. Seven models demonstrated excellent discriminatory abilities in the testing cohort, random forest model showed the highest discriminatory ability (AUC: 0.880, 95% confidence interval(CI): 0.835–0.926). Logistic regression had the best calibration, while random forest exhibited the greatest clinical net benefit. By comparing features across various models, we confirmed the efficacy of traditional indicators like anti-dsDNA antibodies, complement levels, serum creatinine, and urinary red and white blood cells in predicting and distinguishing PLN. Additionally, we uncovered the potential value of previously controversial or underutilized indicators such as serum chloride, neutrophil percentage, serum cystatin C, hematocrit, urinary pH, blood routine red blood cells, and immunoglobulin M in predicting PLN.

**Conclusion:**

This study provides a comprehensive perspective on incorporating a broader range of biomarkers for diagnosing and predicting PLN. Additionally, it offers an ideal non-invasive diagnostic tool for SLE patients unable to undergo renal biopsy.

## 1 Introduction

Systemic lupus erythematosus (SLE) is a chronic autoimmune disease with an unclear etiology, characterized by the loss of normal immune tolerance to endogenous nuclear components (1, 2). The development of lupus nephritis (LN) in SLE patients is multifactorial, involving dysregulation of the complement system, abnormal production of autoantibodies, environmental influences, and genetic factors (3). LN is defined by the deposition of immune complexes within the renal glomeruli, confirmed through histopathological examination. It represents one of the most common and severe organ challenges in SLE patients (4), posing a significant risk factor for morbidity and mortality (5, 6). In 2003, the International Society of Nephrology/Renal Pathology Society (ISN/RPS) classified LN (7), excluding advanced sclerosing LN (Type VI), into proliferative and non-proliferative types based on renal histopathology. Non-proliferative lupus nephritis (NPLN) includes types I, II, and isolated type V, with milder inflammation and renal damage, leading to a favorable prognosis (8). Conventional treatment tends to be conservative (9). Proliferative lupus nephritis (PLN) refers to type III or IV lesions alone or combined with type V lesions (10–12), indicating a more severe condition compared to NPLN, with a significantly increased risk of progression to end-stage renal disease (ESRD) and poor prognosis (13, 14). Due to its detrimental impact on renal function and prognosis (14), the treatment strategy for PLN involves overall immunosuppression and maintenance therapy to control inflammation and autoimmune reactions (9).

Given the differences in treatment strategies and prognosis between PLN and NPLN, rapid diagnosis and early targeted treatment are crucial for improving renal function prognosis, particularly for PLN (9, 15). However, renal biopsy, as the gold standard for diagnosing PLN, is not always feasible or safe due to potential complications (16), technological limitations in primary healthcare facilities (9, 15), and contraindications for certain patients with specific conditions (17). Therefore, the development of a safe, non-invasive diagnostic method is urgently needed.

Currently, research on using big data analysis to predict clinical factors related to PLN is still quite scarce. There is limited evidence demonstrating the potential of biomarker analysis in predicting PLN risk or identifying individuals who may develop PLN at the onset of their disease. Based on this, we have developed and validated various machine learning models to predict the occurrence of PLN. The development of these models is crucial for achieving early diagnosis of PLN in clinical practice and effectively stratifying PLN from NPLN, thereby improving patient prognosis.

## 2 Materials and methods

### 2.1 Study participants

This study was a single-center retrospective study conducted at West China Hospital, Sichuan University, a tertiary teaching hospital. Between 2011 and 2021, a total of 1312 patients diagnosed with SLE with renal involvement underwent renal biopsy.

#### 2.1.1 Inclusion criteria

(1)Patients clinically diagnosed with SLE and renal involvement, with renal involvement manifested by persistent proteinuria (>0.5g protein per day), presence of cellular casts (red blood cells, hemoglobin, granular, tubular, or mixed), urinary protein-to-creatinine ratio >500mg/g (50mg/mmol), or renal dysfunction. (2) Patients who underwent renal biopsy and were pathologically diagnosed with PLN or NPLN according to the 2003 ISN/RPS classification criteria. NPLN includes class I, II, or V LN, while PLN includes class III, IV, or III/IV with V LN (10–12).

#### 2.1.2 Exclusion criteria

**Figure 1:**
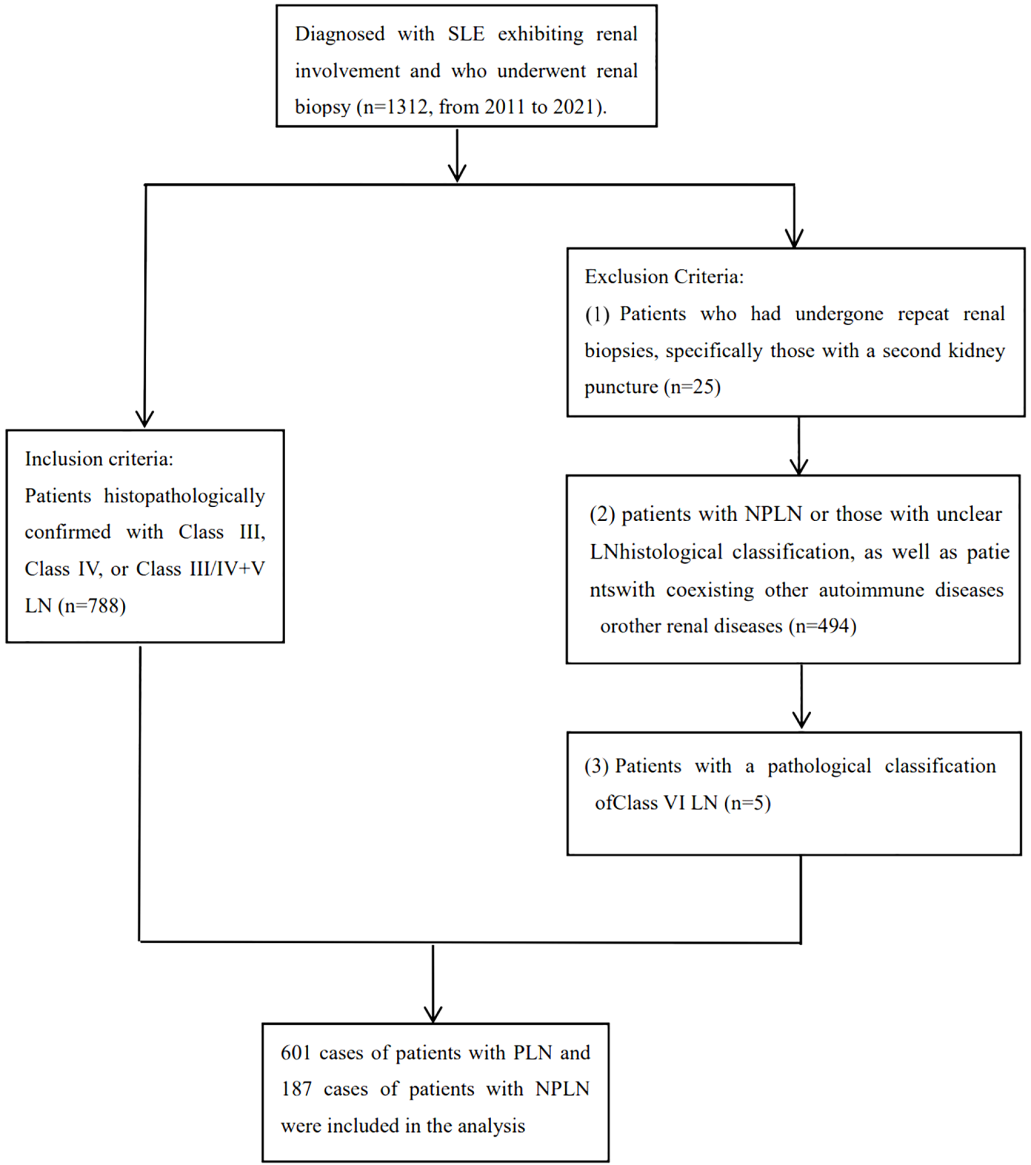
The flow chart for inclusion and exclusion.

(1)Patients with repeat biopsies who underwent clinical intervention between the two biopsy procedures, to ensure model accuracy, patients undergoing their second biopsy were excluded based on the time of renal puncture. (2) Patients with non-LN or unclear pathological diagnosis of LN (such as limited glomerular number in renal biopsy, making classification difficult). (3) Patients with class VI LN or other renal diseases besides LN (such as primary glomerulonephritis, diabetic nephropathy, hepatitis B virus-related nephropathy, drug-induced renal injury, etc.). (4) Patients with concurrent autoimmune diseases such as rheumatoid arthritis, autoimmune hepatitis, ANCA-associated vasculitis, etc. The flow chart for inclusion and exclusion is provided in **Figure 1**.

After confirming subjects based on inclusion and exclusion criteria, we collected clinical and laboratory characteristics. Clinical features included renal biopsy pathology, demographics (age, gender), admission physical exam indicators (systolic and diastolic blood pressure, body mass index, pulse). Laboratory features encompassed all indicators detected during hospitalization (hematology, immunology, biochemistry, coagulation, routine exams, etc.). Data were collected from the most recent data before renal biopsy. Features with <30% missing values for laboratory features and <60% for observation samples were selected. Missing values were then addressed using multiple imputation methods.

### 2.2 Machine learning models overview

This study developed nine models, including generalized linear models such as logistic regression, Least Absolute Shrinkage and Selection Operator(LASSO), ridge regression, and elastic net regression, as well as support vector machines including linear and radial basis kernel functions, and decision tree models such as classical decision trees, conditional inference trees, and random forests. Logistic regression estimates model parameters using Maximum Likelihood Estimation (MLE) (18). LASSO regression, ridge regression, and elastic net regression improve the model by adding an additional shrinkage penalty term to ordinary least squares (OLS). LASSO controls the sum of absolute values of coefficients through L1 regularization, achieving coefficient shrinkage and variable selection, making the final model more concise and interpretable. Ridge regression introduces a penalty term for the sum of squared coefficients through L2 regularization, improving prediction stability and accuracy. However, ridge regression lacks the ability to perform feature selection when dealing with datasets with a large number of features. Elastic net combines L1 and L2 regularization to penalize coefficients in the regression model, enabling feature selection that ridge regression cannot achieve and handling correlations between features that LASSO may overlook (19, 20). These three models using shrinkage penalties can avoid multicollinearity and overfitting problems. Support Vector Machine (SVM) maximizes the margin between two classes by hyperplane (decision boundary) in a high-dimensional feature space to distinguish different classes. Linearly separable SVMs are called linear kernel SVMs, while nonlinearly separable SVMs use kernel tricks to map data to higher-dimensional space for linear separability, known as radial basis kernel SVMs (21). Classical decision trees build tree models based on maximizing purity, conditional inference trees select features and build models based on statistical significance tests, and random forests are an ensemble supervised learning algorithm that constructs multiple decision trees by random sampling of samples and features (22). The final prediction classification of a sample is determined by the most frequently occurring class among the predictions of all trees to improve overall prediction accuracy.

### 2.3 Machine learning models establishment

We randomly split the dataset into training and testing sets in a 7:3 ratio. Machine learning models were built on the training set, with elastic net regression optimizing model parameters using grid search, and the remaining models selecting optimal parameters through ten-fold cross-validation. We chose the point of maximum Youden index as the optimal cutoff value to distinguish between PLN and NPLN.

### 2.4 Models validation

In this study, the ability of the models to differentiate between PLN and NPLN was evaluated using Receiver Operating Characteristic Curve (ROC) on both the training and testing datasets. The Youden index was used to determine the threshold for assessing accuracy, sensitivity, and specificity. Calibration curves were plotted to evaluate the calibration accuracy of the models, ensuring the reliability of their predictive results. To analyze the clinical utility of the models, the study quantified the net benefit of PLN risk probability at different thresholds using Decision Curve Analysis (DCA) curves, thereby determining the clinical application value of the models.

### 2.5 Statistical methods

In the study, continuous data for PLN and NPLN groups in the training and testing sets were represented using median and interquartile range (IQR), and compared using the Wilcoxon rank-sum test (Mann-Whitney U test). Categorical data were presented as frequencies (proportions) and compared using the chi-square test. The logistic regression model included LASSO-selected predictor variables or clinically relevant variables as independent variables, while other models used all predictor variables as independent variables. All models were built with PLN or NPLN as the response variable. Model parameters were selected using ten-fold cross-validation or grid search. The optimal cutoff value for distinguishing PLN and NPLN was determined using the point of maximum Youden index. All statistical tests were two-tailed, with significance set at P < 0.05. Data analysis was conducted using R (version 4.2.2) and RStudio.

### 2.6 Ethics statement

This study was approved by the biomedical research ethics committee of West China Hospital (2022–239). The informed content was waived. The study conformed to the Declaration of Helsinki.

## 3 Results

### 3.1 Study participants

This study enrolled 1312 SLE patients with kidney involvement, of whom 788 met the inclusion and exclusion criteria for analysis. Data on 7 clinical features (pathological classification, age, gender, systolic and diastolic blood pressure, BMI, and pulse) and 1265 laboratory features were collected. After addressing missing values, analysis included 780 patients and 129 features, with PLN or NPLN as the outcome. 6 clinical features and 122 laboratory features (detailed in **Supplementary Material 1**) were considered. Baseline characteristics of the training and testing sets (**Table 1**) showed no significant differences (*P* > 0.05) in age, gender, blood pressure, BMI, and pulse rate. However, significant differences (*P* < 0.05) in blood pressure and 13 other major laboratory features were observed between PLN and NPLN patients in both sets.

**Table 1:** Comparison of patient characteristics in this study.

	Training Cohort					Test Cohort						Missing rates
	NPLN(n=130)		PLN(n=417)		P1	NPLN(n=55)		PLN(n=178)		P2	P3	
Sex	Female	115(88.5)	Female	352(84.4)	0.254	Female	50(90.9)	Female	147 (82.6)	0.135	0.767	0.26%
	Male	15(11.5)	Male	65(15.6)		Male	5(9.1)	Male	31(17.4)			
Age	33.00[25.00, 43.00]		33.00[24.00, 42.00]		0.408	31.00[26.00, 40.00]		31.00[24.00, 41.75]		0.771	0.665	0%
BPS	120.00[109.00,134.00]		135.00[123.00,150.00]		<0.001*	123.00[115.50, 131.00]		133.00[120.00, 148.00]		<0.001*	0.316	8.59%
BPD	80.00[72.00, 87.75]		88.00[79.00, 98.00]		<0.001*	81.00[75.00, 90.00]		86.00[75.25, 97.75]		0.013*	0.475	8.72%
BMI	Lean	27(20.8)	Lean	51(12.2)	0.047*	Lean	3(5.5)	Lean	23(12.9)	0.203	0.659	43.21%
	Normal	63(48.5)	Normal	252(60.4)		Normal	38(69.1)	Normal	99(55.6)			
	Overweight	26(20.00)	Overweight	73(17.5)		Overweight	8(14.5)	Overweight	39(21.9)			
	Obese	14(10.8)	Obese	41(9.8)		Obese	6(10.9)	Obese	17(9.6)			
pulse	80.00[75.00, 98.00]		84.00[78.00, 94.00]		0.702	86.00[79.00, 97.50]		82.00[78.00, 98.00]		0.375	0.220	14.36%
C3	0.6[0.45, 0.81]		0.39[0.26, 0.54]		<0.001*	0.61[0.46, 0.81]		0.39[0.29, 0.56]		<0.001*	0.313	2.56%
C4	0.13[0.08, 0.20]		0.09[0.05, 0.13]		<0.001*	0.14[0.11, 0.21]		0.09[0.05, 0.13]		<0.001*	0.801	6.28%
IGM	1315.00[790.75, 1785.00]		936.00[649.00, 1400.00]		<0.001*	1230[870.00, 1805.00]		987.50[678.00, 1447.50]		0.017*	0.441	11.79%
RBC	4.30[3.95, 4.72]		3.54[3.06, 4.10]		<0.001*	4.26[4.03, 4.73]		3.64[3.19, 4.07]		<0.001*	0.430	0.13%
Cl	105.80[102.73, 107.47]		108.50[104.90, 111.90]		<0.001*	105.40[102.90, 108.20]		108.00[104.93, 110.68]		0.001*	0.378	3.46%
NEUTP	64.55[57.02, 74.72]		72.70[63.50, 80.40]		<0.001*	64.10[57.05, 72.75]		71.20[62.80, 80.18]		0.003*	0.378	1.03%
CysC	1.04[0.86, 1.26]		1.78[1.30, 2.45]		<0.001*	1.09[0.92, 1.40]		1.79[1.23, 2.57]		<0.001*	0.548	0.13%
Cr	55.00[47.58, 63.50]		86.10[62.00, 138.00]		<0.001*	52.00[45.50, 63.65]		91.20[62.00, 153.62]		<0.001*	0.607	0.26%
HCT	0.39[0.35, 0.42]		0.32[0.27,0.36]		<0.001*	0.38[0.36, 0.44]		0.31[0.27, 0.36]		<0.001*	0.903	0.13%
UPH	6.50[6.00, 7.00]		6.00[6.00, 6.50]		<0.001*	6.50[6.00, 7.00]		6.00[6.00, 6.50]		0.002*	0.241	1.79%
URBC	Normal	37(28.5)	Normal	30(7.2)	<0.001*	Normal	15(27.3)	Normal	12(6.7)	<0.001*	0.795	1.92%
	High	93(71.5)	High	387(92.8)		High	40(72.7)	High	166(93.3)			
UWBC	Normal	87(66.9)	Normal	120(28.8)	<0.001*	Normal	32(58.2)	Normal	60(33.70)	0.001*	0.666	1.79%
	High	43(33.1)	High	297(71.2)		High	23(41.8)	High	118(66.3)			
Anti-dsDNA	Normal	99(76.2)	Normal	130(31.2)	<0.001*	Normal	44(80.0)	Normal	76(42.7)	<0.001*	0.013*	4.23%
	High	31(23.8)	High	287(68.8)		High	11(20.0)	High	102(57.3)			

For the comparison of characteristics between the two cohorts, PLN and NPLN, continuous variables are presented as median [IQR], and categorical variables are presented as Frequency (proportion). P1 represents the comparison between PLN and NPLN in the training cohort, P2 represents the comparison between PLN and NPLN in the test cohort, and P3 represents the comparison between the training and test cohorts. P-value < 0.05 (*) indicates a statistically significant difference. In the table, BPS and BPD represent systolic blood pressure and diastolic blood pressure, respectively; RBC, HCT, and NEUTP represent red blood cells, hematocrit, and the percentage of neutrophils in whole blood, respectively; C3, C4, IgM, Cl, CysC, Cr, and Anti-dsDNA represent serum levels of complement 3, complement 4, immunoglobulin M, chloride, cystatin C, creatinine, and anti-double-stranded DNA antibodies, respectively; UPH, URBC, and UWBC represent PH, red blood cells, and white blood cells in urine, respectively.

### 3.2 Machine learning models establishment

**Figure 2:**
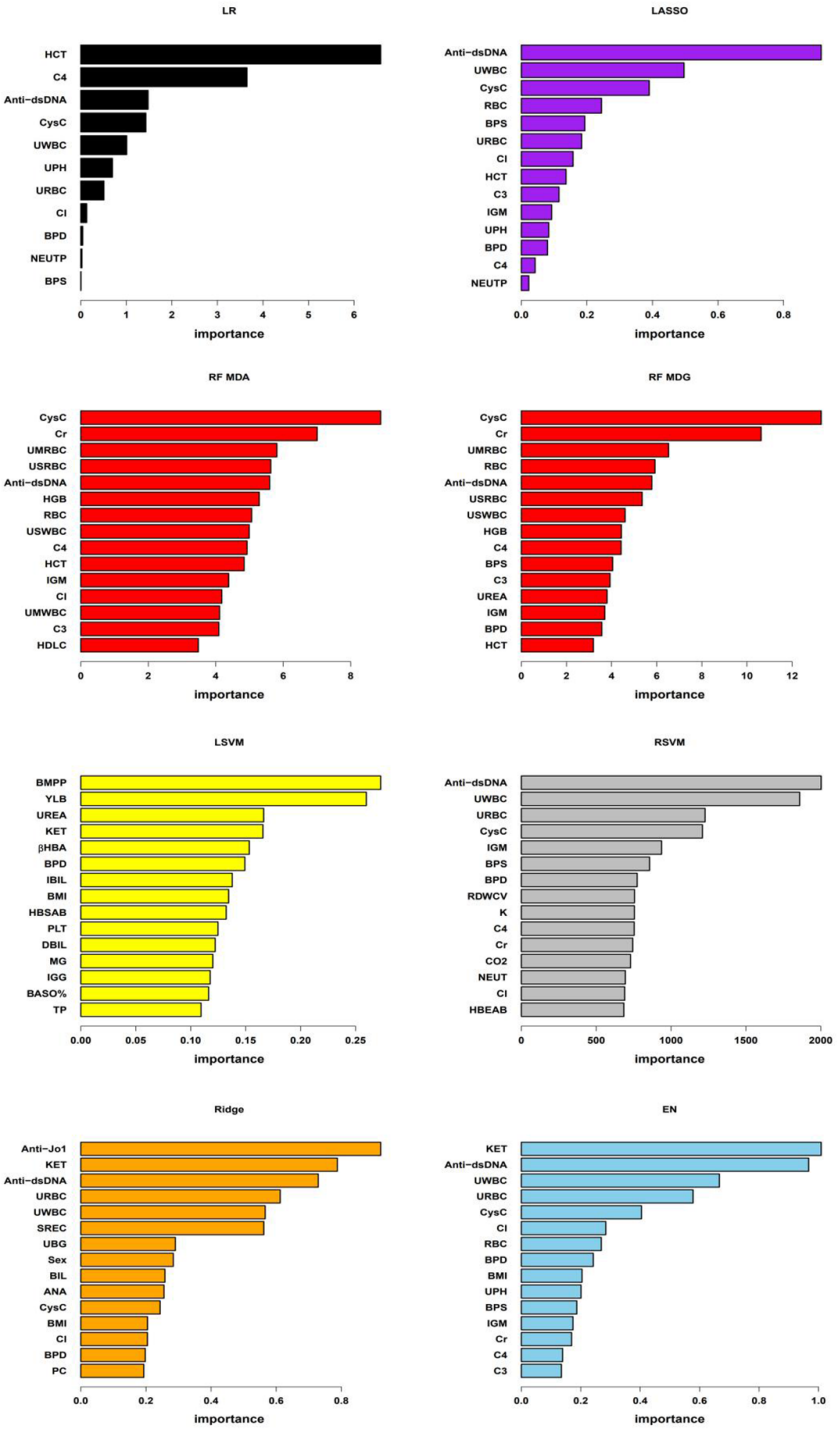
Important features of the models As shown in the figure, “LR” denotes logistic regression model, “RF MDA” and “RF MDG” represent random forest model’s variable importance assessed by MeanDecreaseAccuracy and MeanDecreaseGini respectively, “LSVM” stands for linear kernel Support Vector Machine model, “RSVM” denotes radial kernel Support Vector Machine model, “LASSO”, “Ridge”, and “EN” respectively represent Least Absolute Shrinkage and Selection Operator, Ridge regression, and Elastic Net regression models. In the LR, LSVM, LASSO, Ridge, and EN models, variable importance is assessed based on the coefficients of each variable within the models. For the RSVM model, the importance of each feature is determined by the average contribution of that feature across all support vectors. In RF model, variable importance is evaluated using MeanDecreaseAccuracy and MeanDecreaseGini. Due to the differing importance of features across various models and the different methods used to assess this importance, the specific importance values of each feature vary between models in the figure. In the figure, BPS and BPD represent systolic blood pressure and diastolic blood pressure, respectively; RBC, HCT, HGB, PLT, BASO%, RDWCV, NEUT and NEUTP represent red blood cells, hematocrit, hemoglobin, platelet count, basophil percentage, red blood cell distribution width CV, neutrophil absolute count and the percentage of neutrophils in whole blood, respectively; C3, C4, IgM, Cl, CysC, Cr, HDLC, UREA, BMPP, βHBA, IBIL, HBSAB, DBIL, MG, IGG, TP, K, CO2, HBEAB, Anti-Jo1, ANA and Anti-dsDNA represent serum levels of complement 3, complement 4, immunoglobulin M, chloride, cystatin C, creatinine, high-density lipoprotein cholesterol, urea, bactericidal membrane permeability protein, beta-hydroxybutyrate, indirect bilirubin, hepatitis B surface antibody, direct bilirubin, magnesium, immunoglobulin G, total protein, potassium, carbon dioxide binding, hepatitis B e antibody, Anti-Jo1 antibody, antinuclear antibody and anti-double-stranded DNA antibodies, respectively; UPH, URBC, UWBC, KET, SREC,UBG, BIL and PC represent PH, red blood cells, white blood cells, ketones, small round epithelial cells, urobilinogen, bilirubin and pus cells in urine, respectively. UMRBC, USRBC, USWBC, and UMWBC represent urinary sediment microscopy red blood cells, urinary sediment red blood cells, urinary sediment white blood cells, and urinary sediment microscopy white blood cells, respectively.

The logistic regression model utilized ten-fold cross-validation with LASSO variable selection, identifying 11 non-zero potential predictor variables at a lambda value of 0.04171. The classical decision tree model, through ten-fold cross-validation, determined 4 terminal nodes with a complexity parameter of 0.04615385, involving features such as Serum Cystatin C (CysC), anti-double stranded DNA antibodies (Anti-dsDNA) and urinary red blood cells (URBC). The conditional inference tree model considered only four variables: hematocrit (HCT), Anti-dsDNA, systolic blood pressure (BPS), and CysC. In the random forest model, the optimal number of trees corresponding to the minimum error rate was 169. Variable importance was assessed using MeanDecreaseAccuracy and MeanDecreaseGini. The linear kernel support vector machine (LSVM) model explored 21 different cost parameters, with optimal selection achieved at 0.01 through ten-fold cross-validation. The radial basis kernel support vector machine (RSVM) model, utilizing 441 parameter combinations of cost and gamma, identified the optimal combination: gamma of 0.0001 and cost of 100. The LASSO model employed ten-fold cross-validation, selecting a lambda of 0.04171 and identifying 14 non-zero variables. For the ridge regression model, ten-fold cross-validation determined the optimal lambda as 0.0899. The elastic net model used cross-validation to select optimal alpha and lambda, with alpha at 0.2894737 and lambda at 0.03757956. Except for the classical decision tree model and the conditional inference tree model, the features or the top 15 important features for the remaining models are listed in **Figure 2**.

### 3.3 Models validation

**Figure 3:**
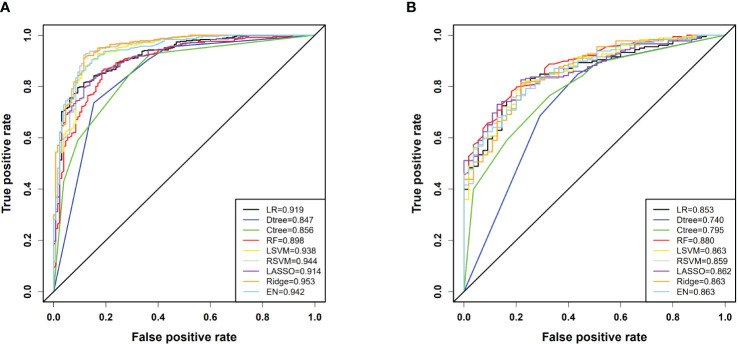
**(A)** ROC curves for each model in the training cohort; **(B)** ROC curves for each model in the testing cohort. In the figure, LR denotes Logistic Regression, Dtree represents Classic Decision Tree, Ctree stands for Conditional Inference Tree, RF is Random Forest, LSVM indicates Linear Kernel Support Vector Machine, RSVM denotes Radial Kernel Support Vector Machine, LASSO stands for Least Absolute Shrinkage and Selection Operator, Ridge refers to Ridge Regression, and EN signifies Elastic Net.

In our model training set, all models achieved an AUC exceeding 0.8, indicating strong classification performance. Notably, the ridge regression model stood out with an impressive AUC of 0.953 [95% confidence interval(CI): 0.933, 0.973]. In the testing set, except for the classical decision tree and conditional inference tree, all models maintained AUC above 0.8, with the random forest model performing the best (AUC: 0.880 [95% CI: 0.835, 0.926]). RSVM exhibited the highest sensitivity in the training set (0.923 [95% CI: 0.893, 0.945]), while logistic regression showed the best specificity (0.908 [95% CI: 0.844, 0.948]). Additionally, RSVM achieved the highest accuracy (0.914 [95% CI: 0.887, 0.935]). In the testing set, ridge regression ranked first in sensitivity (0.837 [95% CI: 0.775, 0.885]), while logistic regression had the highest specificity (0.818 [95% CI: 0.695, 0.900]). The ridge regression model also led in accuracy (0.803 [95% CI: 0.747, 0.849]). The differentiation performance of each model in the training and testing cohorts is illustrated in **Figure 3** and **Table 2**.

**Table 2:** Comparison of each model’s performance in terms of AUC, sensitivity, specificity, and accuracy in the training and testing cohorts.

Training Cohort	Threshold	Sensitivity[95CI]	Specificity[95CI]	Accuracy[95CI]	AUC[95CI]
LR	0.808	0.796[0.755,0.832]	0.908[0.844,0.948]	0.823[0.788,0.852]	0.919[0.894,0.945]
Dtree	0.830	0.736[0.692,0.776]	0.846[0.774,0.899]	0.762[0.725,0.796]	0.847[0.808,0.887]
Ctree	0.784	0.849[0.811,0.880]	0.708[0.624,0.779]	0.815[0.781,0.846]	0.856[0.820,0.892]
RF	0.662	0.861[0.824,0.891]	0.815[0.739,0.873]	0.850[0.818,0.878]	0.898[0.867,0.929]
LSVM	0.752	0.880[0.845,0.908]	0.885[0.817,0.930]	0.881[0.851,0.906]	0.938[0.912, 0.963]
RSVM	0.704	0.923[0.893,0.945]	0.885[0.817,0.930]	0.914[0.887,0.935]	0.944[0.920,0.969]
LASSO	0.726	0.823[0.783,0.856]	0.862[0.791,0.911]	0.832[0.798,0.861]	0.914[0.887,0.940]
Ridge	0.684	0.911[0.880,0.935]	0.885[0.817,0.930]	0.905[0.877,0.927]	0.953[0.933,0.973]
EN	0.677	0.904[0.872,0.929]	0.854[0.782,0.905]	0.892[0.863,0.916]	0.942[0.920,0.964]
Test Cohort	Threshold	Sensitivity	Specificity	Accuracy	AUC[95CI]
LR	0.808	0.747[0.678,0.806]	0.818[0.695,0.900]	0.764[0.705,0.814]	0.853[0.801,0.904]
Dtree	0.830	0.685[0.614,0.749]	0.709[0.578,0.813]	0.691[0.629,0.747]	0.740[0.664,0.815]
Ctree	0.784	0.764[0.696,0.821]	0.673[0.541,0.782]	0.742[0.683,0.795]	0.795[0.733,0.858]
RF	0.662	0.815[0.751,0.865]	0.709[0.578,0.813]	0.790[0.733,0.837]	0.880[0.835,0.926]
LSVM	0.752	0.764[0.696,0.821]	0.800[0.675,0.886]	0.773[0.714,0.822]	0.863[0.813,0.913]
RSVM	0.704	0.803[0.738,0.855]	0.727[0.597,0.828]	0.785[0.728,0.833]	0.859[0.809,0.910]
LASSO	0.726	0.775[0.708,0.831]	0.782[0.655,0.872]	0.777[0.719,0.826]	0.862[0.814,0.910]
Ridge	0.684	0.837[0.775,0.885]	0.691[0.559,0.798]	0.803[0.747,0.849]	0.863[0.811, 0.914]
EN	0.677	0.820[0.757,0.870]	0.709[0.578,0.813]	0.794[0.737,0.841]	0.863[0.814,0.912]

LR denotes Logistic Regression, Dtree represents Classic Decision Tree, Ctree stands for Conditional Inference Tree, RF is Random Forest, LSVM indicates Linear Kernel Support Vector Machine, RSVM denotes Radial Kernel Support Vector Machine, LASSO stands for Least Absolute Shrinkage and Selection Operator, Ridge refers to Ridge Regression, and EN signifies Elastic Net. Threshold represents the optimal cutoff value determined based on the Youden’s index, and sensitivity, specificity, and accuracy are determined based on this Threshold.

**Figure 4:**
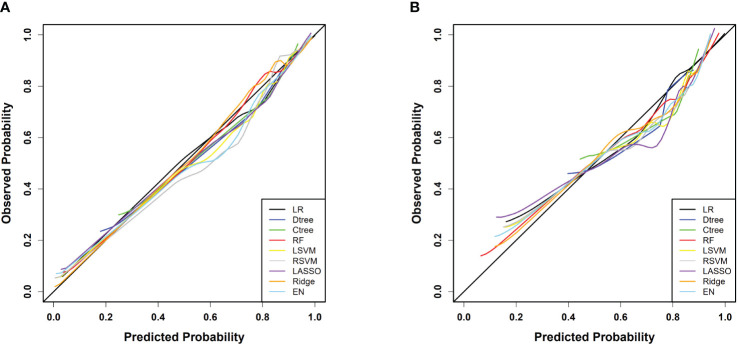
**(A)** Calibration curves for each model in the training cohort; **(B)** Calibration curves for each model in the testing cohort.

Calibration curve analysis indicated good consistency between predicted values and actual observations for all models. Particularly, in the training set, the ridge regression model demonstrated the highest prediction accuracy, with a mean squared error (MSE) of only 0.00011, highlighting its precision in fitting the dataset. Furthermore, in the testing set, the logistic regression model exhibited the best performance with an MSE value of 0.00080, showcasing its strong generalization ability on independent datasets. **Figure 4** and **Table 3** reflect the model’s prediction accuracy performance for the two cohorts.

**Table 3:** Comparison of calibration performance of each model in the training and testing cohorts.

Training Cohort	MAE	MSE	0.9 QAE	Test Cohort	MAE	MSE	0.9 QAE
LR	0.014	0.00043	0.034	LR	0.018	0.00080	0.051
Dtree	0.029	0.00148	0.063	Dtree	0.027	0.00142	0.062
Ctree	0.037	0.00163	0.050	Ctree	0.057	0.00420	0.084
RF	0.008	0.00014	0.019	RF	0.027	0.00096	0.046
LSVM	0.014	0.00051	0.038	LSVM	0.036	0.00207	0.070
RSVM	0.016	0.00083	0.041	RSVM	0.045	0.00274	0.082
LASSO	0.024	0.00093	0.062	LASSO	0.052	0.00418	0.106
Ridge	0.007	0.00011	0.018	Ridge	0.031	0.00136	0.064
EN	0.019	0.00080	0.043	EN	0.042	0.00235	0.067

MAE is the model’s mean absolute error of prediction, MSE is the model’s mean squared error of prediction, and 0.9 QAE is the 90% of Quantile of Absolute Error for the model.

**Figure 5:**
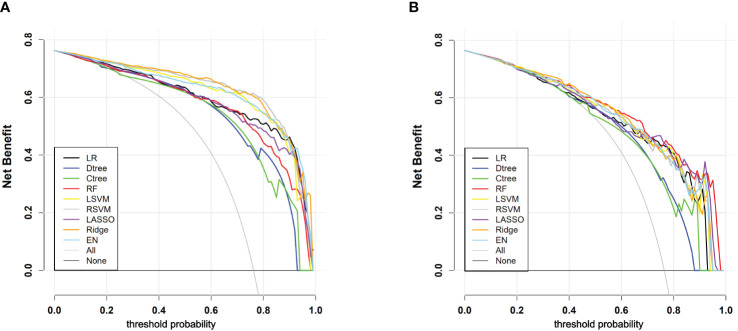
**(A)** DCA curves for each model in the training cohort; **(B)** DCA curves for each model in the testing cohort. “All” signifies that all patients have PLN and have all received an intervention, which resulted in a net benefit for the patients; “None” means that all patients have NPLN, none have received an intervention, and the net benefit is zero.

Through DCA, we assessed the net benefit performance of the models across various threshold probabilities. In the analysis of the training set, the ridge regression model exhibited a net benefit exceeding the extreme curve, with the broadest range of threshold probabilities. At the optimal threshold, this model achieved the maximum net benefit of 0.628. Similarly, in the testing set, the random forest model’s net benefit surpassed the extreme curve, with the widest interval of threshold probabilities, reaching the highest value of 0.520 at the optimal threshold probability point. Overall, most models demonstrated significant net benefits in practical decision support, except for classical decision tree and conditional decision tree models. **Figure 5** and **Table 4** illustrate the models’ value for clinical applications.

**Table 4:** Comparison of DCA performance of each model in the training and testing cohorts.

Training Cohort	LR	Dtree	Ctree	RF	LRVM	RSVM	LASSO	Ridge	EN
Probability Range	0.03- 0.99	0.18- 0.92	0.25- 0.93	0.03- 0.98	0.01- 0.98	0.01- 0.98	0.03- 0.98	0.01- 0.98	0.01- 0.98
Threshold NB	0.512	0.383	0.395	0.567	0.576	0.623	0.517	0.628	0.614
Test Cohort	LR	Dtree	Ctree	RF	LRVM	RSVM	LASSO	Ridge	EN
Probability Range	0.19- 0.92	0.40- 0.87	0.45- 0.89	0.04- 0.97	0.13- 0.93	0.11- 0.94	0.10- 0.96	0.10- 0.94	0.10- 0.94
Threshold NB	0.390	0.188	0.263	0.520	0.429	0.452	0.465	0.478	0.488

Within this Probability Range, the net benefit of the model exceeds that of the extreme curves. Threshold NB is the net benefit of the model when the threshold probability is set to the value determined by Youden’s index.

## 4 Discussion

SLE is a potentially life-threatening autoimmune disease, with PLN being one of its most severe clinical manifestations, significantly increasing the risk of patient mortality and renal failure (13, 14). While renal biopsy remains the gold standard for diagnosing PLN, its invasiveness, potential risks, and inapplicability in specific circumstances limit its widespread use, particularly for certain special conditions or contraindicated patients. This limitation underscores the urgent need for a non-invasive diagnostic approach. An exhaustive search of the PubMed database reveals a scarcity of studies using machine learning to predict the risk of PLN. Consequently, this study aimed to harness high-dimensional feature data to construct and validate a series of machine learning models, aiming to accurately predict the risk of PLN occurrence.

In this study, we observed a stable overall prevalence rate of 76% for PLN. To our knowledge, only two previous studies attempted to construct predictive models for PLN. In these two studies, one model achieved a maximum AUC of 0.84 in the training set and 0.82 in the validation set (23), while the other study reported a lower predictive accuracy of only 0.637 (24). In comparison, our study utilized a larger dataset to build models, and the results demonstrate that our models achieved a maximum AUC of 0.953 in the training set and AUCs exceeding 0.850 in the testing set for all models except classical decision tree and conditional inference tree. Regarding predictive accuracy, our training set performance ranged from 0.823 to 0.914, while the testing set ranged from 0.764 to 0.803. Although the performance of models may be influenced by the selection of predictive variables, considering the scale of data and number of predictive variables used in our study surpass previous research, our models outperform those constructed in previous studies in all aspects. Furthermore, among the various machine learning models we developed, they all demonstrated high consistency and predictive accuracy. In clinical decision-making, except for classical and conditional decision tree models, all other models showed significant net benefits, validating not only the efficacy of the models but also enhancing their practical value in assisting clinical decision-making. Furthermore, the study observed a statistical difference in Anti-dsDNA between the training and testing cohorts. First, since the data was randomly split, we cannot guarantee identical distributions between the training and testing sets, making such differences possible. Second, the testing data is used to evaluate the model’s performance. In real-world applications, the testing cohort represents the patients we aim to predict, and it is unlikely to find a dataset with a distribution identical to that of the training cohort. Lastly, the AUC for all seven models in the testing set is greater than 0.85, indicating that the models perform well even with discrepancies in data distribution, further demonstrating their strong generalization ability. Additionally, in both cohorts, the positive rate of Anti-dsDNA in PLN is significantly higher than in NPLN, which is consistent with the model’s conclusions. Therefore, our model is not affected by this factor.

In this study, we evaluated seven predictive models with AUC values exceeding 0.85 in the testing set. The results showed that among these high-performing models, at least three models consistently identified 16 key predictive factors. These factors cover various physiological and biochemical indicators, specifically including BPS, diastolic blood pressure (BPD), serum chloride (Cl), neutrophil percentage (NEUTP), CysC, HCT, complement 4 (C4), urine pH (UPH), URBC, urinary white blood cells (UWBC), Anti-dsDNA, serum creatinine (Cr), red blood cell count in the blood (RBC), immunoglobulin M (IGM), complement 3 (C3), and BMI. The majority of shared features had a data missing rate of less than 5%, with blood pressure data missing rates of 8.59% and 8.72%, and C4 missing rate of 6.28%, all within the range of 5%-10%. However, the missing rate for IGM reached 11.79%, and the BMI’s missing rate was significantly higher than other variables at 43.21%. This suggests that although BMI as a research indicator has certain potential value, its high data missing rate requires further exploration and validation in future studies. All seven models consistently demonstrated the importance of blood pressure; six models highlighted the significance of CysC, URBC, UWBC and Anti-dsDNA; C4 was considered a significant factor in five models; while IGM was identified as a key variable in four models. It is noteworthy that blood pressure, URBC, UWBC, Anti-dsDNA, C3 and C4, and Cr are not only traditionally used laboratory markers for predicting LN but also demonstrated their ability to distinguish between PLN and NPLN in this study. Furthermore, these biomarkers predicting PLN are consistent with those identified in previous studies (23, 24), further validating the stability and reliability of these indicators.

**Figure 6:**
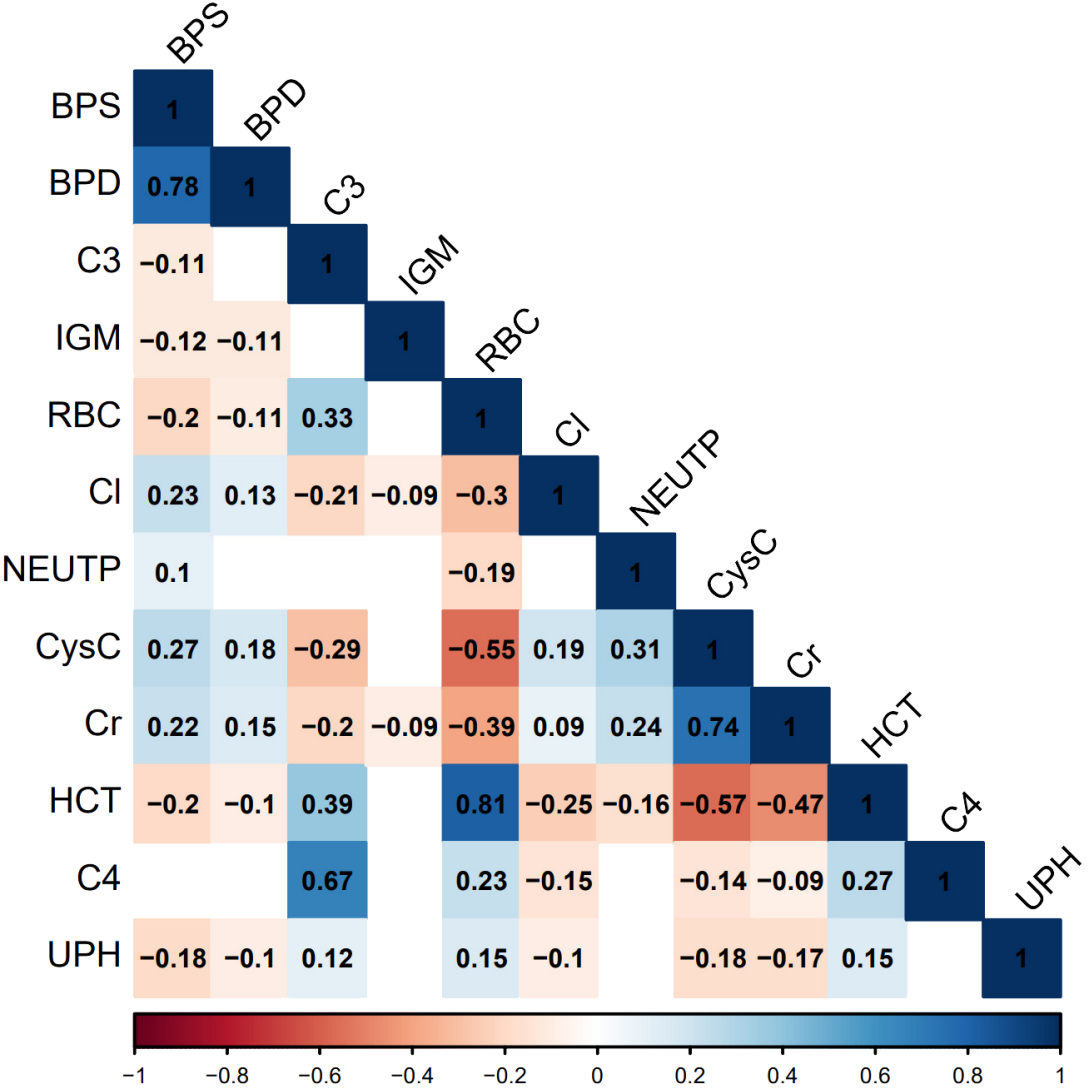
Correlation graph of common important features. Statistical significance is determined at P<0.05. In the figure, non-significant correlations are represented as blank spaces. Significant correlations are displayed in blue or red, with specific correlation values shown. Blue indicates positive correlations, red indicates negative correlations, and the color intensity reflects the strength of the correlation.

Although previous studies have revealed a significant correlation between CysC levels and the severity and pathological grades of LN (25, 26), the specific connection between it and PLN remains insufficiently supported by empirical evidence. The exact association between neutrophils and PLN is also subject to controversy (27, 28). Anemia symptoms in LN patients may be related to renal damage and the generation of autoantibodies (29, 30), however, there is currently no in-depth research indicating a direct link between anemia symptoms and PLN. Recent research indicates that IgM deposited in LN glomeruli can activate the complement system, driving disease progression, and lower IgM levels in LN patients’ serum may be associated with more severe manifestations of the disease (31). LN patients may experience electrolyte and acid-base balance disturbances due to renal impairment (32), manifested by elevated serum Cl levels and decreased UPH. This study further clarifies some previously disputed or less widely used indicators, such as CysC, NEUTP, HCT, RBC, IGM, UPH, and Cl, indicating their potential importance in predicting PLN. These findings underscore the need for greater attention to these indicators in clinical practice. The identification of consensus indicators in this study not only highlights their crucial role in predicting PLN but also provides strong clues for future research on PLN biomarkers. Additionally, the correlation analysis of common features indicates a strong positive correlation between serum cystatin C and creatinine, systolic and diastolic blood pressure, red blood cells and hematocrit, as well as complement 3 and complement 4. Conversely, cystatin C or creatinine show a strong negative correlation with red blood cells or hematocrit (**Figure 6**). These findings are consistent with the clinical presentations of the patients and the characteristics listed in **Table 1**.

While our study has certain significance, there are limitations. It is a single-center retrospective study, and the results have not been validated through multicenter studies due to the relative rarity of lupus nephritis patients and limitations in research resources. Therefore, before translating the models into clinical practice, it is necessary to further validate and refine our models using multicenter data from different ethnic backgrounds. Additionally, considering data integrity, the study excluded non-routine testing variables with a missing rate exceeding 30%, which may result in the models not fully capturing all potential important explanatory features.

Our study pioneers the analysis of detailed, high-dimensional data from lupus nephritis patients over the past decade, encompassing comprehensive clinical and laboratory examination data. Multiple machine learning models were developed and comprehensively evaluated, affirming their discriminative ability, accuracy calibration, and potential clinical application. Beyond classical decision tree and conditional inference tree models, the other models demonstrate strong overall performance, offering innovative non-invasive methods for diagnosing and predicting PLN. Moreover, they show promise as reliable supplements or even alternatives to renal biopsy, especially in LN stratified management, crucial for patients ineligible for renal biopsy. Additionally, by identifying common features, this study suggests considering a more comprehensive panel of biomarkers for PLN diagnosis and prediction. At the clinical level, physicians can select the most suitable model based on patient-specific conditions and treatment needs, enhancing the accuracy of early detection and intervention for PLN. Our research significantly enhances the technical capabilities for early PLN diagnosis and treatment, providing clinicians with more robust and refined auxiliary tools.

## Data availability statement

The original contributions presented in the study are included in the article/**Supplementary Materials**, further inquiries can be directed to the corresponding authors.

## Ethics statement

This study was approved by the biomedical research ethics committee of West China Hospital (2022-239). The informed content was waived. The study conformed to the Declaration of Helsinki. The studies were conducted in accordance with the local legislation and institutional requirements. Written informed consent for participation was not required from the participants or the participants’ legal guardians/next of kin because This was a retrospective study using only historical clinical data from patients.

## Author contributions

PY: Conceptualization, Data curation, Formal analysis, Investigation, Methodology, Validation, Writing – original draft, Writing – review & editing. ZL: Conceptualization, Data curation, Formal analysis, Validation, Writing – original draft. FL: Conceptualization, Formal analysis, Writing – original draft. YS: Conceptualization, Formal analysis, Writing – original draft. PL: Formal analysis, Writing – original draft. QZ: Formal analysis, Writing – original draft. KW: Methodology, Writing – original draft. XZh: Methodology, Writing – original draft. XZe: Formal analysis, Project administration, Resources, Supervision, Validation, Writing – review & editing. YW: Data curation, Methodology, Project administration, Resources, Supervision, Validation, Writing – review & editing.
