# Supplementary material for: Machine learning models predicts risk of proliferative lupus nephritis
Source: Front Immunol. 2024 Jun 11;15:1413569. doi: 10.3389/fimmu.2024.1413569 (PMC11196753; doi:10.3389/fimmu.2024.1413569)
Supplement: Supplementary file 1 [file DataSheet_1.pdf]

Supplementary Material 1

The 122 laboratory features used for model training

Test Group Item Name	Sub-Test Item Name	English Abbreviation
Blood Cell Analysis	Lymphocyte Percentage	LYMPH%
Blood Cell Analysis	Basophil Percentage	BASO%
Blood Cell Analysis	Absolute Basophil Count	BASO
Blood Cell Analysis	Monocyte Percentage	MONO%
Blood Cell Analysis	Mean Corpuscular Hemoglobin Concentration	MCHC
Blood Cell Analysis	Absolute Monocyte Count	MONO
Blood Cell Analysis	Absolute Lymphocyte Count	LYMPH
Blood Cell Analysis	Red Blood Cell Distribution Width Coefficient of Variation	RDWCV
Blood Cell Analysis	Red Blood Cell Distribution Width Standard Deviation	RDWSD
Blood Cell Analysis	Absolute Neutrophil Count	NEUT
Blood Cell Analysis	Mean Corpuscular Hemoglobin	MCH
Blood Cell Analysis	White Blood Cell Count	WBC
Blood Cell Analysis	Mean Corpuscular Volume	MCV
Blood Cell Analysis	Eosinophil Percentage	EO%
Blood Cell Analysis	Hematocrit	HCT
Blood Cell Analysis	Hemoglobin	HGB
Blood Cell Analysis	Platelet Count	PLT
Blood Cell Analysis	Absolute Eosinophil Count	EO
Blood Cell Analysis	Red Blood Cell Count	RBC
Blood Cell Analysis	Neutrophil Percentage	NEUTP
ENA Antibody Spectrum	Anti-Sm Antibody	Anti-SM
ENA Antibody Spectrum	Anti-Rib Antibody	Anti-RIB
ENA Antibody Spectrum	Anti-Jo-1 Antibody	Anti-Jo-1
ENA Antibody Spectrum	Anti-Scl-70 Antibody	Anti-Scl-70
ENA Antibody Spectrum	Anti-RNP Antibody	Anti-RNP
ENA Antibody Spectrum	Anti-SS-A Antibody	Anti-SSA
ENA Antibody Spectrum	Anti-SS-B Antibody	Anti-SSB
Complement	Complement C3	C3
Complement	Complement C4	C4
Complement	Properdin Factor B	PFB
Anti-Neutrophil Cytoplasmic Antibodies screening	Lactoferrin	LACT
Anti-Neutrophil Cytoplasmic Antibodies screening	Elastase	ELA
Anti-Neutrophil Cytoplasmic	Cathepsin G	CATHG

Antibodies screening		
Anti-Neutrophil Cytoplasmic Antibodies screening	Anti-Neutrophil Cytoplasmic Antibodies	ANCA
Anti-Neutrophil Cytoplasmic Antibodies screening	bactericidal membrane permeability protein	BMPP
Anti-Nuclear Antibodies	Anti-double stranded DNA Antibody	Anti-dsDNA
Anti-Nuclear Antibodies	Anti-Nuclear Antibodies	ANA
Anti-Neutrophil Cytoplasmic Antibodies	Myeloperoxidase	MPO
Anti-Neutrophil Cytoplasmic Antibodies	Proteinase 3	PR3
Rheumatoid Factor	Rheumatoid Factor	RF
Immunoglobulin GAM	Immunoglobulin M	IGM
Immunoglobulin GAM	Immunoglobulin G	IGG
Immunoglobulin GAM	Immunoglobulin A	IGA
Pre-Transfusion Full Set	Hepatitis B Surface Antigen	HBSAG
Pre-Transfusion Full Set	Hepatitis B Surface Antibody	HBSAB
Pre-Transfusion Full Set	Hepatitis B e Antigen	HBEAG
Pre-Transfusion Full Set	Hepatitis B e Antibody	HBEAB
Pre-Transfusion Full Set	Hepatitis B Core Antibody	HBCAB
Pre-Transfusion Full Set	Hepatitis C Antibody	HCVAB
Pre-Transfusion Full Set	HIV Antigen-Antibody Combo Test	HIV
Total IgE	Immunoglobulin E	IGE
Routine Coagulation Tests	Prothrombin Time	PT
Routine Coagulation Tests	Thrombin Time	TT
Routine Coagulation Tests	Thrombin Time Ratio	TTR
Routine Coagulation Tests	Activated Partial Thromboplastin Time	APPT
Routine Coagulation Tests	Activated Partial Thromboplastin Time Ratio	APTTR
Routine Coagulation Tests	International Normalized Ratio	INR
Routine Coagulation Tests	Fibrinogen	FIB
Biochemistry Tests	Direct Bilirubin	DBIL
Biochemistry Tests	Gamma-Glutamyl Transferase	GGT
Biochemistry Tests	Total Protein	TP
Biochemistry Tests	Globulin	GLB
Biochemistry Tests	Alkaline Phosphatase	ALP
Biochemistry Tests	Creatine Kinase	CK
Biochemistry Tests	Lactate Dehydrogenase	LDH
Biochemistry Tests	Cholesterol	CHOL

Biochemistry Tests	Serum Cystatin C	CysC
Biochemistry Tests	Total Bilirubin	TBIL
Biochemistry Tests	Hydroxybutyrate Dehydrogenase	HBDH
Biochemistry Tests	Uric Acid	UA
Biochemistry Tests	Albumin	ALB
Biochemistry Tests	Triglycerides	TG
Biochemistry Tests	Glucose	GLU
Biochemistry Tests	Low-Density Lipoprotein Cholesterol	LDLC
Biochemistry Tests	Indirect Bilirubin	IBIL
Biochemistry Tests	High-Density Lipoprotein Cholesterol	HDLC
Biochemistry Tests	Alanine Aminotransferase	ALT
Biochemistry Tests	Aspartate Aminotransferase	AST
Biochemistry Tests	Urea	UREA
Biochemistry Tests	AST/ALT	A/A
Biochemistry Tests	Albumin/Globulin Ratio	A/G
Biochemistry Tests	Anion Gap	AG
Biochemistry Tests	Natrium	NA
Biochemistry Tests	Inorganic Phosphorus	PO4
Biochemistry Tests	Calcium	CA
Biochemistry Tests	Kalium	K
Biochemistry Tests	Magnesium	MG
Biochemistry Tests	Serum Beta-Hydroxybutyrate	βHBA
Biochemistry Tests	Carbon Dioxide Binding Power	CO2
Biochemistry Tests	Chloride	Cl
Biochemistry Tests	Creatinine	CREA
Urinalysis Chemical Analysis	White Blood Cell	UWBC
Urinalysis Chemical Analysis	Urobilinogen	UBG
Urinalysis Chemical Analysis	Nitrites	NIT
Urinalysis Chemical Analysis	Specific Gravity	SG
Urinalysis Chemical Analysis	Acidity/Alkalinity	UPH
Urinalysis Chemical Analysis	Red Blood Cell	URBC
Urinalysis Chemical Analysis	Urine Glucose	UGLU
Urinalysis Chemical Analysis	Ketones	KET
Urinalysis Chemical Analysis	Urine Bilirubin	BIL
Urinalysis Chemical Analysis	Urine Protein	PRO
Urinary Sediment Analysis	White Blood Cell	USWBC
Urinary Sediment Analysis	Conductivity	CONDUCTIVI
Urinary Sediment Analysis	Casts	CAST
Urinary Sediment Analysis	Epithelial Cells	EC
Urinary Sediment Analysis	Red Blood Cell	USRBC
Urinary Sediment Analysis	Pathological Casts	PACAST
Urinary Sediment Analysis	Bacteria	BACT

Urinary Sediment Microscopy	granular Casts	UMGCAST
Urinary Sediment Microscopy	Mucus Threads	MUCUS
Urinary Sediment Microscopy	General epithelial cells	GEC
Urinary Sediment Microscopy	Red Blood Cell	UMRBC
Urinary Sediment Microscopy	Pus Cells	PC
Urinary Sediment Microscopy	White Blood Cell	UMWBC
Urinary Sediment Microscopy	small round epithelial cells	SREC
Urinary Protein Quantification	24-hour Urine Protein	24hUPO
Urinary Protein Quantification	24-hour Urine Volume	24hUO
Urinary Protein Quantification	Microprotein	UMP
Fecal routine	Red Blood Cell	FRBC
Fecal routine	Yeast-like Cells	YLB
Fecal routine	White Blood Cell	FWBC
Fecal Occult Blood Test	Occult Blood	FBLD
